# Inside or outside: Evaluation of the efficiency enhancement of OLEDs with applied external scattering layers

**DOI:** 10.1038/s41598-019-54640-x

**Published:** 2019-12-09

**Authors:** Pen Yiao Ang, Paul-Anton Will, Simone Lenk, Axel Fischer, Sebastian Reineke

**Affiliations:** Dresden Integrated Center for Applied Physics and Photonic Materials (IAPP), TU Dresden, Nöthnitzer Str. 61, 01187 Dresden, Germany

**Keywords:** Engineering, Electrical and electronic engineering, Materials science, Materials for optics, Lasers, LEDs and light sources, Optics and photonics, Applied optics, Lasers, LEDs and light sources, Optical materials and structures, Optical physics, Optical techniques, Applied physics, Characterization and analytical techniques, Physics, Techniques and instrumentation

## Abstract

Improving the efficiency of organic light-emitting diodes (OLEDs) by enhancing light outcoupling is common practise and remains relevant as not all optical losses can be avoided. Especially, externally attached scattering layers combine several advantages. They can significantly increase the performance and neither compromise the electric operation nor add high costs during fabrication. Efficiency evaluations of external scattering layers are often done with lab scale OLEDs. In this work we therefore study different characterization techniques of red, green and blue lab scale OLEDs with attached light scattering foils comprising TiO_2_ particles. Although we observe an increased external quantum efficiency (EQE) with scattering foils, our analysis indicates that areas outside the active area have a significant contribution. This demonstrates that caution is required when efficiency conclusions are transferred to large area applications, for which effects that scale with the edges become less significant. We propose to investigate brightness profiles additionally to a standard EQE characterizations as latter only work if the lateral scattering length is much smaller than the width of the active area of the OLED. Our results are important to achieve more reliable predictions as well as a higher degree of comparability between different research groups in future.

## Introduction

Organic light-emitting diodes (OLEDs) have been established in industry and are nowadays found in many smartphones and large TV panels. To open further markets a constant development is needed regarding costs, lifetime and efficiency. The latter is mainly limited by the inherent high refractive index of the materials, which leads to optical confinement of photons in the planarized multi-layer structure^1,2^. Generated light may be waveguided in organic thin films, transparent anodes or substrates. Over the years many different light outcoupling strategies have been developed, which can be grouped in internal and external solutions.

Internal light outcoupling structures are built inside of the OLEDs and have direct influence on the photon propagation. Typical examples are periodic gratings^3–6^, low refractive index grids^7,8^ or more random structures in form of bucklings^9,10^ or scattering particles between substrate and electrode^11–14^. While the internal structures can result in high efficiency improvement, a careful implementation must be done to ensure electrical stability of the OLEDs. Another possibility is to directly embed scattering structures in the substrate^15–18^.

In contrast, external light outcoupling structures are simply attached to the outer surface of the substrate. This leads to reduced reflection at the glass-air interface. Although the external structures can only extract substrate modes, they represent an easily applicable and cheap way to enhance OLED efficiency and can even be combined with internal structures. Typical examples of external light outcoupling structures are micro-lenses^19–23^ or flat scattering layers including particles^24–27^, air voids^28,29^ and crystallized organic layers^30^. In combination with high-refractive index substrates, extraordinary efficiencies can be reached^31^. Most important, external outcoupling approaches do not alter the electrical system and can thus be easily transferred and applied to any OLED architecture.

However, a superior method that works generally for all emission colors and device types has not been found. The sheer amount of possibilities slows down the search for the ideal light outcoupling structure and so far there is no approach that is frequently reproduced or that is focused on in literature. While optical simulations are being developed to ultimately predict optimal scattering structures^32,33^, the experimentalists are lacking of standardized characterizations methods in order to make their results comparable. Recent work suggests that also a new metric is needed for defining the enhancement by optical outcoupling structures in general^34^.

For solar cells, the community is vividly discussing the substantial influence of the actual measurement procedures of lab size devices on the performance metrics^35^. For example, masks are used to shield the solar cells from absorbing photons outside of the active area. Similar issues should be considered if external light outcoupling structures lead to inhomogeneous light emission from lab size OLED samples. To the best of our knowledge, however, there is no literature that discusses characterization methods of OLEDs with scattering layers.

In this report, we focus on carefully quantifying the light outcoupling of OLEDs comprising external scattering layers for the case of finite size devices where edge effects cannot be ignored. We study OLEDs with external light scattering foils and discuss the contradicting change in device efficiency resulting from various measurement methods. For that reason, we attach scattering foils to red, green and blue OLEDs. First, the optical characteristics of the foils are presented. Then we compare six measurement methods of OLEDs with the attached foils on laboratory scale (~mm^2^) and validate one method with a larger OLED (~cm^2^). Finally, we propose a method to investigate lab scale OLEDs with external scattering foils.

## Results

### Optical characterization of external light scattering foils

The 2.5 cm × 2.5 cm foils consist mainly of an optical adhesive (NOA63, Norland Products Inc.). The manufacturing process follows a method described by Park *et al*.^36^. Here, a mixture of the adhesive, acetone, and optionally high-refractive-index scattering particles is spin-coated on a glass substrate. After curing, foils of an approximate thickness of 30 μm can be peeled off the substrate. A plain foil without particles serves as reference “NOA63”. The second foil includes TiO_2_ scattering particles with a diameter of 50 nm “NOA 63:TiO_2_ (unmilled)”. For the third sample “NOA 63:TiO_2_ (milled)”, the TiO_2_ particles are milled with ZrO_2_ particles beforehand, which prevents cluster formation of the TiO_2_ particles. The ZrO_2_ particles are then removed and not added to the mixture.

**Figure 1:**
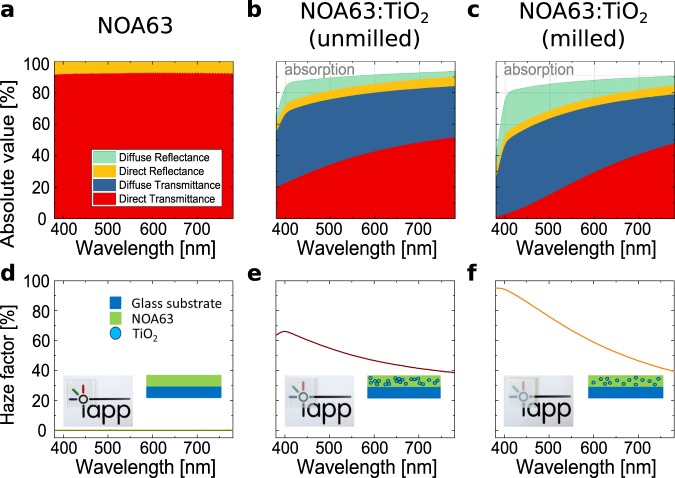
Measured optical properties of the scattering foils on top of a glass substrate: (**a**,**d**) NOA63 (**b**,**e**) NOA63:TiO_2_ (unmilled) (**c**,**f**) NOA63:TiO_2_ (milled). The insets show photographs of the films on top of the IAPP logo and schematic illustrations of the film composition.

Figure 1(a–c) shows the measured transmittance, reflectance and absorption under perpendicular incidence of light for the three foils attached to a glass substrate by index matching oil. The transmittance and reflectance is further divided into direct and diffuse parts, where *direct* means that the light propagation remains in the same straight line as the incident light, i.e. directly reflected rays hit the light source again. Diffuse parts take into account all angles different from the initial and directly reflected one, and thus, represent an integrated measure of one or multiple scattering events. The sum of direct and diffuse components result in the total value. For the reference foil NOA63 on glass, no light scattering can be observed as there are only direct components. Its average total transmittance in the visible range of the spectrum (380 to 780 nm) is 92%, which is as transparent as glass (≈ 92%). Therefore, it can be assumed that the adhesive mixture does not lead to noteworthy changes in light propagation. In contrast, the foils with scattering particles redirect a significant part of the incident light leading to diffuse components. The average total transmittance decreases to 78% and 67% with insertion of unmilled and milled TiO_2_ particles, respectively. This can be attributed to occurring absorption and diffuse reflectance, which are both highest for the milled TiO_2_ particles. Compared to the reference, the direct transmittance strongly drops, but the direct reflectance only slightly decreases indicating similar surface morphologies and we assume that the direct reflectance mainly originates from the first interface between air and NOA63 which is the same for all three layers. The scattering must therefore come mainly from the bulk of the foils.

Figure 1(d–f) shows the calculated haze, given by the ratio of the diffuse to the total transmitted light. Haze is typically used to quantify how strong a film scatters light. Accordingly, images behind hazy foils appear laterally broadened and milky. With higher haze the appearance becomes more blurry as demonstrated by the inset photographs of Fig. 1. Although the average diffuse transmittance is rather similar for the unmilled (38%) and milled (42%) particle mixtures, the latter shows much higher haze due to a lower direct transmittance. For both samples the haze generally increases towards lower wavelengths, which is an expected behaviour for Rayleigh scattering. The foil with milled TiO_2_ particles reaches haze values of more than 90%. However, haze provides neither information about the total transmittance nor about the angular dependence of the scattered light. For example, the haze values above 90% of NOA63:TiO_2_ (milled) have a total transmittance of less than 30%.

**Figure 2:**
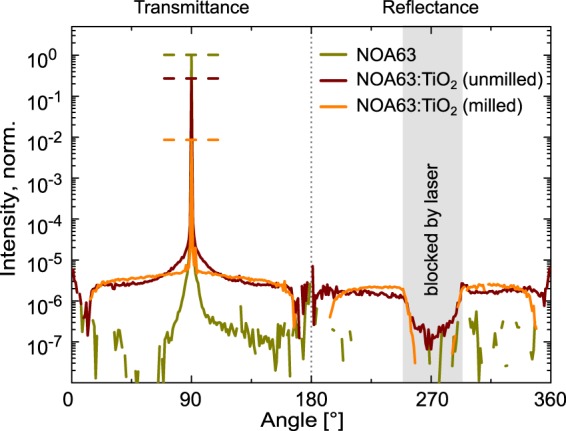
Recorded light intensity around the scattering foils under vertical irradiation of a 405 nm laser. While NOA63 shows mostly direct transmittance, NOA63:TiO_2_ (milled) exhibits most uniform scattering in all direction.

To get a measure of the scattering directionality, we illuminate the foils with a laser under a normal incidence and rotate the fixed arrangement of foil and laser while recording the scattered light with a stationary photodiode. The laser has a wavelength of $\lambda =405\,{\rm{nm}}$ for which the haze factor differs most between the three samples with 0%, 66%, and 94%, respectively. Figure 2 shows the recorded light intensity over the entire rotation for the three foils. All values are normalized to the peak of the reference NOA63 at 90°, which represents the direct transmission of the laser emission. For the angles from 0° to 180° the light is transmitted through the foils and from 180° to 360° the light is reflected. At around 270° the direct reflectance is blocked by the laser arrangement. The reference foil NOA63 shows mostly direct transmittance, since the intensity peak is narrow and intensities at angles different from 90° quickly reach the resolution limit of the setup as described in the Experimental section. The ratio of the peak heights of Fig. 2 correspond in general to the direct transmittance measurements at $\lambda =405\,{\rm{nm}}$ of Fig. 1. Also in accordance with the prior optical characterization, both samples with TiO_2_ particles exhibit diffuse transmittance and reflectance. This is shown by the two orders of magnitude higher intensity compared to the noise level of the reference. For the foil with milled TiO_2_ particles there is an almost constant intensity for all angles different from 90° and 270°. This indicates that all the incident light being perpendicular to the surface of the foil (coming from 270°), is redistributed relatively evenly in all other directions if not directly transmitted at 90° or reflected back to 270°.

### Comparison of characterization methods of lab scale OLEDs with attached foils

**Figure 3:**
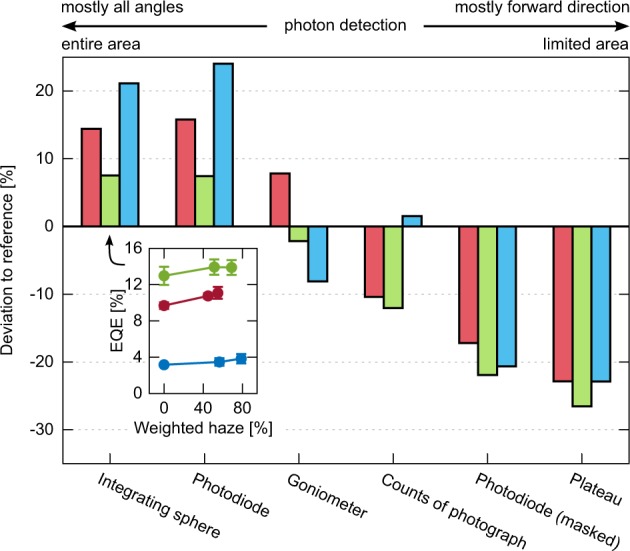
Deviations of various measurements of red, green, and blue bottom-emitting OLEDs with the attached NOA63:TiO_2_ (milled) scattering foil compared to the NOA63 reference. The inset shows the EQE at *j* = 15 mA/cm^2^ over the spectrally weighted haze.

**Figure 4:**
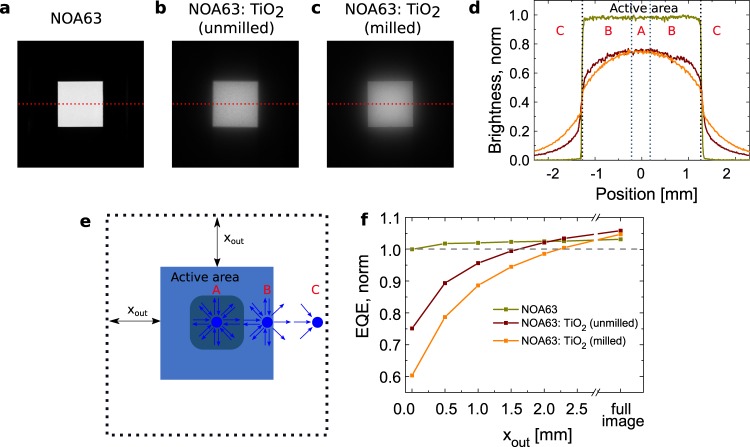
(**a**–**c**) Photographs of blue lab scale OLEDs with the three attached foils in grey scale. (**e**) Classification scheme for three distinct emission regions (A, B, C) and integration area depending on *x*_out_. (**d**) Intensity cross sections extracted along the red dashed lines of the photographs. The regions are indicated for unmilled TiO_2_. (**f**) Integrated counts of photographs depending on square integration area. All images are taken with the same geometry and identical camera settings. The settings were adjusted to ensure that the brightest image is not saturated, which is proven by remaining signature of noise of the cross sections. For discussion the reader is referred to the main text.

**Figure 5:**
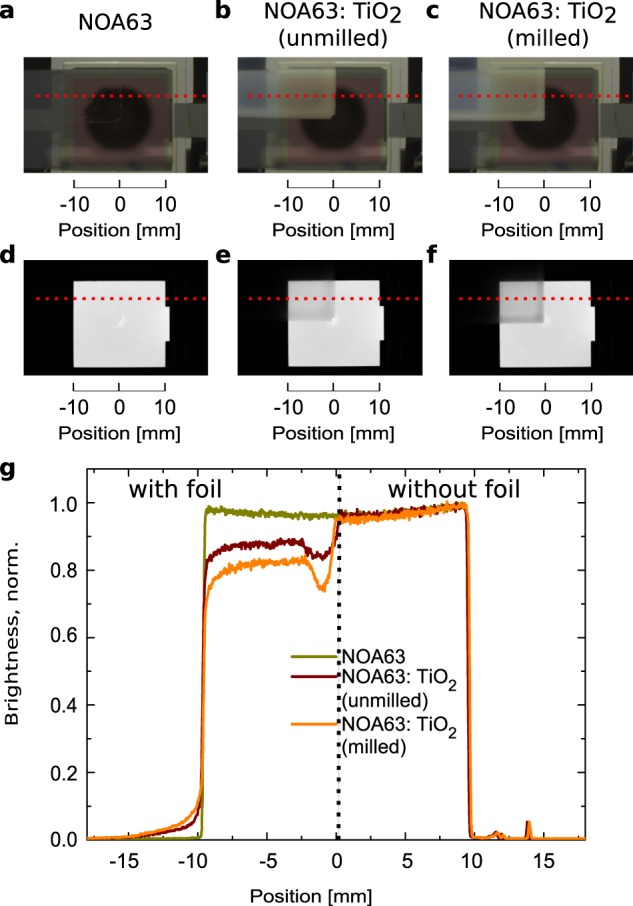
External scattering foils attached to the corner of a larger red OLED with an active area of approximately 2 cm × 2 cm. (**a**–**c**) Photographs of the OLEDs with the foils attached to the top left corner and being switched off. The red dashed lines indicate the position of the extracted brightness profiles used for (**g**). (**d**–**f**) Gray scale photographs of OLEDs under electrical operation.

It is often observed that the EQE of OLEDs with scattering layers increases with the haze of the outcoupling films^24,37,38^. To test this hypothesis, we attached the three foils with index-matching oil to red, green, and blue bottom-emitting (RGB) OLEDs and measured the EQE in an integrating sphere. The inset of Fig. 3 shows indeed an approximate proportionality between EQE and the spectrally weighted haze for all emission colors. The milled TiO_2_ particles yield increased EQE values for the red, green and blue OLEDs of +14.4%, +7.5%, and +21.1% compared to the NOA63 reference. This might be a bit surprising since the scattering foils exhibit increasing total reflectance as demonstrated in Fig. 1. But according to Bathelt *et al*.^25^ a scattering enhancement is a trade-off between back-reflection at the substrate-air interface and backscattering at particles. The back-reflection within the substrate cannot be directly measured, but due to the observed EQE improvement, we assume that the total reflection from glass to air is reduced with insertion of scattering particles. Figures 4(a–c) and 5(a–c) demonstrate, however, that we cannot reproduce the observation of increased brightness in photos of OLEDs with attached scattering foils. This is in contrast to photographs reported by Preinfalk *et al*.^39^, although the optical properties of our scattering foils are almost the same as in the report. From the enhanced EQE we would also expect enhanced brightness.

To study the contradictory results, we measured a set of identical samples with six different methods. We compare the red, green, and blue bottom-emitting OLEDs with the NOA63 foil as reference to the same OLEDs with applied foil possessing the highest haze (NOA63:TiO_2_ milled). All of the methods show different deviations to the reference as demonstrated in Fig. 3. In the following we briefly explain the principle of each method and compare the obtained deviations.

The first measurement with an integrating sphere is a widely accepted standard for measuring absolute photon numbers^40^. Here, the substrate edges of the OLEDs are covered and all photons being emitted in the forward hemisphere are collected. We find enhanced EQE with application of the foils for all colors with +14.4%, +7.5%, and +21.1%.

For the second measurement, a photodiode ($\varnothing =10\,{\rm{mm}}$) records the emitted light at a fixed distance of approximately 8 cm above the sample. This method does not cover all emission angles, but it receives light from the entire sample area that is larger then the actual emitting area. We find similar results as obtained by the integrating sphere. The trends and the values are almost reproduced for all samples.

**Figure 6:**
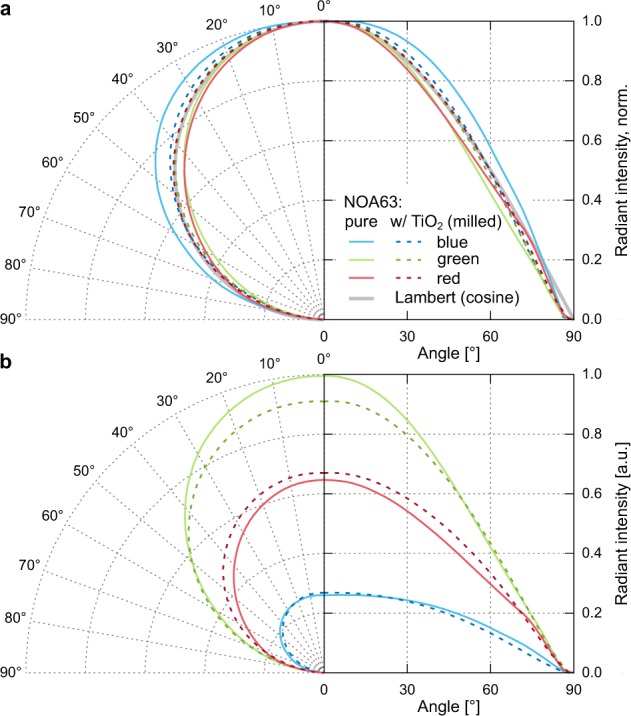
(**a**) Normalized radiant intensity and (**b**) radiant intensity of RGB bottom-emitting OLEDs with reference foil NOA63 and scattering foil NOA63:TiO_2_ (milled). A Lambert emitter is indicated with a cosine function as the detection cone is larger than the extend of the emitting area.

Third, the EQE can also be calculated from spectro-goniometer measurements under assumption of azimuthal symmetry^41^. Figure 6 shows the angular dependent radiant intensity of the red, green, and blue OLEDs with and without milled particles in the NOA63 foil. The result of the sample with unmilled TiO_2_ particles is not shown as it lies in between the other two samples. The foils with scattering particles lead to an emission characteristics very close to one of a Lambert emitter for all colours, also supported by Fig. 2 where scattering particles lead to a more homogeneous scattering in all directions. But now the EQE deviations differ from the two previous methods. For red the radiant intensity increases, but the EQE gains only +7.8%. For green, the EQE slightly drops by −2.1%. Figure 6(b) suggests that this is caused by a decreased forward emission. The EQE for the blue OLED surprisingly drops by −8.1%, which is in stark contrast to previous measurements. Here, the forward emission is slightly increased, but emission at higher angles is reduced. Our goniometer setup has a detection cone that covers the entire quadratic active area. Therefore, it also detects light from areas that are outside the active area and the surroundings consist of different electrode pathways, neighbouring active areas, and the encapsulation glass. The precise angular distribution from each different spot cannot be measured. Thus, the measured signal has to be understood as an averaged value from the outside and inside active area along one azimuthal angle.

Fourth, to study the sole emission of the active area, we performed measurements with the photodiode again. However, this time we covered the active area of the OLEDs with a circular mask ($\varnothing =2\,{\rm{mm}}$) and all detected light is from forward emission with emission angles equal or smaller 4°. The high haze scattering foil leads to decreased photo-current of approximately −20% for all colors. The perceived brightness from the front view is thus clearly reduced, which has already been seen qualitatively in Fig. 4(a–c). Again, the decline is in conflict to the goniometer measurements, where for red and blue the forward emission was enhanced.

As a fifth technique, we take images of the lab scale OLEDs and integrate the intensity of all pixels in a certain area. Figure 4(f) shows exemplarily the integrated value for the blue OLED as a function of integration boundary in form of a square around the emitting pixel. Within the active area (${x}_{{\rm{out}}}=0$) the OLED with scattering foil reach only 60% of the reference value. The integrated counts of the full image, however, lead to a slight enhancement for blue with +1.5%. The full image value of red and green is reduced by approximately −10%.

Lastly, the image cross sections are taken for comparison. Therefore, the maximum plateau value in region A of the Fig. 4(e) is extracted for each color. The highest haze scattering foil leads to a drop of more than −20% for all colors. The plateau values agree relatively well to the masked photodiode measurements. Further discussion of Fig. 4 is found in section Lateral scattering: Emission from outside the active area as cause of measurement discrepancy.

In summary, depending on the measurement we find enhanced or reduced efficiency values for OLEDs with attached external scattering foils. There are two methods each that provide similar results. The integrating sphere and the photodiode measurement result in enhancements of up to +24%, whereas the plateau and the masked photodiode measurement show a decrease by −27%. The outcome of the goniometer measurements and the integrated counts of the photographs lies in between. The photograph analysis will be repeated for the large area OLEDs in the next section.

### Photographs of larger scale OLEDs with attached foils

For lighting applications, OLEDs with larger areas are required. To test potential use of the foils, we attach them to a larger red OLED with an active area of approximately 2 cm × 2 cm. Figure 5(a–c) show photographs of the foil that is only attached in the top left region of the active area in daylight. The round black shade in the center is the reflection of the camera lens. In Fig. 5(a) the edge of the foil is hardly visible, in b) and c) the foils appear milky white. The second row of Fig. 5 shows the same configuration under OLED illumination in a dark environment so that the difference each foils makes can be seen within one photo. Foils with scattering particles clearly reduce the brightness. The edge of the foils additionally appears darker due to an enhanced thickness from the spin-coating process. Extracted brightness profiles along the red dashed lines are shown in Fig. 5(g). The left half is emission through the glass substrate and foil while the right half is only the emission through the glass substrate as reference. There is no difference to pure glass if NOA63 is attached which proves its high transparency once again (c.f. Fig. 1). By inserting scattering particles, the plateau of the brightness between −8 mm and −2 mm (“with foil”) drops. The higher the haze of the scattering the foil, the less light is emitted towards the camera. Therefore, the decreased forward emission is not only found for lab sized OLEDs, but also for large area OLEDs. This suggests that the application of the foils does not lead to efficiency enhancement of the large area samples, although an EQE enhancement for lab sized samples is found by standard characterization setups such as an integrating sphere.

## Discussion

### Lateral scattering: Emission from outside the active area as cause of measurement discrepancy

The seemingly contradiction between enhanced EQE and reduced brightness in photographs, is most notable for the blue lab scale OLED (*cf*. Fig. 3): The decreased forward emission is documented by the masked photodiode measurement, the plateau analysis, and further by the photographs of the OLED with large active area. To explain an enhancement of the integrated values (integrating sphere data) despite the reduced forward emission, one would assume the emission at high angles to be strongly increased becoming a super Lambertian angular pattern. But the goniometer measurement even shows the opposite: The emission at high angles decreases and the corresponding calculated EQE also drops. For the red and green lab scale OLEDs with effectively different haze, similar logical flaws can be constructed. Device degradation as explanation can be excluded due to repetitive measurements.

All this leads to the conclusion, that the photon emission from outside the active area must have a non-negligible effect on the EQE. These additional photons cannot be completely detected with restricted areas and limited angular ranges and only count entirely in the integrating sphere. The measurement by the photodiode seems to be just as sensitive to the outside emission. In fact, for both measurements the geometry outside of the active area is open to the detection. The light collection geometry of the photograph arrangement is also similar to that of the photodiode measurements.

To test the hypothesis of enhanced EQE by laterally scattered photons, we now analyse the photographs of lab scale OLEDs. Figure 4(d) illustrates a classification of three distinct regions (A, B, C). In region A, a brightness plateau is achieved. Here, each point in the foils scatters light uniformly to the side, but also receives light from neighbouring points so that net exchange is zero. In region B, more light is distributed than received, which leads to a brightness decrease towards the edge of the active area. In region C, light can only be received from the active area and the intensity drops with increasing distance. Figure 4(e) shows that this brightness “decay” has a characteristic length in the range of millimetres. Approximately the same length is also needed to reach a brightness plateau. If the pixel size is similar to twice the decay length, the plateau cannot be reached. This seems to be the case for NOA63 with milled TiO_2_. We assume that the plateau could be slightly higher if the active area would be larger, but we doubt that the final plateau value will rise above the reference. Therefore, it is questionable weather upscaling of the same OLED stack and foils will still lead to EQE enhancements.

Figure 4(f) demonstrates that the integrated counts of lab scale OLEDs with scattering foils are outcompeting the reference when the integration area is large enough. From Fig. 4(a–c) alone, this transition does not seem intuitive, but the area from which counts are collected increases quadratically with the distance. The small contributions over large areas can give a significant fraction to the overall EQE. Both foils outperform the reference and following the trend in Fig. 4(f) the NOA63:TiO_2_ (milled) might even have the highest value for larger photographs.

### Drawing the right conclusions from lab scale OLEDs

Our results show the complexity of OLED characterisation with external scattering foils. Various measurement methods can lead to contradicting conclusions regarding possible efficiency enhancements of scattering foils. We found the EQE enhancement of lab scale OLEDs to be caused by photons that are coupled out more efficiently outside the active area. For large area OLEDs those edge effects will become less significant. This could lead to wrong conclusions of external scattering layer studies, which aim to improve lighting applications.

Similar issues are known for lab scale solar cells. Charges, which are generated outside of the active area due to light absorption, can still be collected by conductive functional layers. Consequently, the device efficiency is overestimated. Therefore, solar cells are typically masked to have a defined area of illumination.

Masking lab scale OLEDs would hinder the determination of the absolute efficiencies, but external scattering layers could be evaluated without the influence of edge effects. For transferring results from small lab scale to large area OLEDs, we therefore propose to evaluate the brightness plateau, e.g. by taking camera images at exactly the same acquisition parameters. Please note that the postprocessing of the images must not alter the linearity between light intensity and pixel value which can be tested by taking a series of images at different exposure times (“reciprocity”). To reach the true plateau, the width of the active area must be larger than the characteristic lateral scattering length. If the external scatting layer shows higher plateau values and has similar angular emission characteristics, then the large area OLED will also be more efficient.

In this work, we see that the plateau value for lab scale devices decreases by about 25% (s. Fig. 3, Plateau) while the angular dependence has not changed much. Therefore, we can assume that the scattering foils, investigated here, would eventually shrink the performance of large area OLEDs which is also supported by the findings for the OLED with larger active area (s. Fig. 5), showing a reduction of the plateau by 15% to 20% for the foil with the highest haze.

## Conclusion

External scattering foils are often investigated to enhance the light outcoupling efficiency of OLEDs. In this work, we find a strong discrepancy between different standard characterization methods. For red, green and blue OLEDs we measure enhanced EQEs with an integrating sphere, but the actual brightness in frontal photographs decreases. We find that whenever photons are emitted from outside the active area, they can have a significant impact on the finally measured EQE. As a result, efficiency enhancements seen for lab scale OLEDs cannot necessarily be achieved in large area applications. These discrepancies between lab scale and final application-relevant device sizes, which are possible both in form of under- and overestimations, need to be minimized to avoid wrong research and development directions. All publications that investigate lab-scale samples with standard methods (e.g. integrating sphere) are basically affected to a certain extent if they do not distinguish between outcoupling inside and outside the active area.

To evaluate external scattering layers with lab scale OLEDs, we propose to study brightness profiles together with angular emission characteristics. First, a brightness plateau must be achieved by having dimensions of active areas larger than the characteristic lateral scattering length. Second, the influence of the angular emission must be quantified. If the plateau value increases and the angular emission is equal, an efficiency enhancement can be expected for large area applications. Only by rethinking the currently used standards, it will be possible to compare scientific results across a variety of different scattering technologies and sample geometries. While the discussion presented in this work was based on OLEDs solely, it can equally be transferred to emerging thin film systems like quantum dot or perovskite LEDs, as they share the same optical architecture to a large extend.

## Experimental

### Sample preparation

In a first step, NOA 63 purchased from Norland Products, US is diluted with acetone in a ratio of 15:1 during an ultrasonic bath for 3 minutes. This solution is used for the first sample “NOA63”. The solution for the second sample additionally contains 2.5 wt% of TiO_2_ nano-particles of 50 nm diameter. The solution for the third sample is made by adding ZrO_2_ particles for milling clustered TiO_2_ nano-particles. The solutions with added particles are alternately put into a planet rotary machine and an ultrasonic bath each 30 minutes in total for 3 hours. Before spin-coating, the solution for the second and the third sample are filtered with Iso-Disc syringe tip filters with a pore size of 0.45 μm in order to have the same processing for both solutions and to remove the ZrO_2_ particles from the third solution. All samples are made by spin coating under 1000 rpm with 30 s revolution time and 30 s hold time on top of a 2.5 cm × 2.5 cm glass substrate. The foils are cured under UV light for 30 minutes and then peeled off for further measurements. The TiO_2_ nanoparticles have a diameter of 50 nm diameter (purchased from mkNANO, Canada). The ZrO_2_ particles are purchased from NETZSCH-Feinmahltechnik GmbH, Germany. All of the three scattering foils have a thickness of around 30 μm, determined with a profilometer Veeco Dektak 150.

### Measurements

Foils are attached to a glass substrate by using an index matchin oil Zeiss Immersol 518F having a refractive index of 1.52 at room temperature. The refractive index of NOA63 is 1.56 and our glass substrate has a refractive index of 1.51 to 1.52 in the visible spectrum range. Transmission and reflectance spectra are measured with an UV-VIS-NIR spectrophotometer MPC 3700 from Shimadzu. For all measurements, the incident light hits the surface of the scattering foil first. Direct transmittance: Transmitted light with a pathway perpendicular to the substrate measured by the spectrometer after taking a baseline. Direct reflectance: A measurement of the reflected light is not possible at perpendicular incidence. The spectrometers therefore takes the reflectance at an angle that is 5° off to perpendicular incidence and we assume that the direct reflectance is almost the same as the one at 5°. Diffuse transmittance: The sample is placed in front of the integrating sphere of the spectrometer, so that all diffuse transmitted light and the direct transmitted light is collected by the integrating sphere. We derive the diffuse transmittance by subtracting the previously determined direct transmittance from the total transmittance. Diffuse reflectance: The sample is placed at one port with the surface of interest pointing towards the integrating sphere of the Shimadzu spectrometer. The incident light ray enters the integrating sphere through a second opposite port. Again, the incident angle is not exactly perpendicular to the surface of the sample, so that also the directly reflected light is collected by the integrating sphere. The light that is scattered back by the scattering foil into the integrating sphere is the total reflected light. We derive the diffuse reflectance after substracting the direct reflectance. The integrating sphere to measure EQE is a model LMS-100 from Labsphere Inc. and the substrate holder of the OLED is white outside and black inside in order to absorb the light that is not directly emitted into the integrating sphere, i.e. backscattered light. The calibration of the integrating sphere has been done including the holder system. Measurements of the forward emission are done with a photodiode SM1PD1A from Thorlabs at a distance of 8 cm to the substrate. The goniometer is a home-built setup consisting of a rotation motor, an adjustment laser and an Ocean Optics spectrometer USB4000. Camera images are taken with a Basler acA1920-40uc that is equipped with a Fujifilm Fujinon HF25XA-1 and a RICOH 20 mm extension tube. The f-number is set to 1.6 and the distance between sample and lense is about 1 cm. The exposure time is 1 ms for blue and green OLEDs, and 2 ms for red OLEDs. The linearity of the camera sensor and the underlying image processing has been tested for reciprocity by taking images at different exposure times. To achieve this, we use a gamma factor of 1. We multiply the brightness of all images that are compared with each other by a global constant factor without saturating the images. Thus, the best range of an 8-bit image file is used by simultaneously retaining the relative brightness ratios and ensuring the best contrast to the reader. The measurement of the 360° angle dependent scattering is done using a laser model STAR405F10 from Roithner Lasertechnik, Austria with a wavelength of 405 nm. The scattered light is measured with a photodiode read out by a source-measuring unit Keithley 2450 at 0 V. The current offset of 1.8 pA, translating into an intensity offset after normalization of 6.5 × 10^−7^. This offset current that is measured if the laser is switched off, is substracted from all measured currents. The noise level of the current is about one order of magnitude lower in the range of 0.1 pA which translates into a noise of the intensity after normalization of 3.6 × 10^−8^. Measurements are done using the software SweepMe! (sweep-me.net).

### OLED fabrication

The lab size devices have an active area of ca. 2.5 mm × 2.5 mm whereas the substrate is 10 times larger in each direction. The glass substrates have pre-structured indium tin oxide (ITO) electrode with a thickness of 90 nm acting as anode. All other thin films are evaporated by thermal evaporation under high vacuum in a chamber system from Kurt J. Lesker Company. 30 nm of 2,2′,7,7′-Tetra(N,N-di-p-tolyl)amino-9,9-spirobifluorene doped with 2,2′-(perfluoronaphthalene-2,6-diylidene)dimalononitrile (Spiro-TTB:F_6_TCNNQ at 2 wt%) is used as hole transport layer. N,N’-di(naphthalen-1-yl)- N,N′-diphenyl-benidine (NPB) is used as electron blocking layer with a thickness of 10 nm. 2-methyl-9,10-bis(naphthalen-2-yl)anthracene doped with 2,5,8,11-Tetra-tert-butylperylene (MADN:TBPe at 1.5 wt%) is used as emitter layer in case of the blue OLED. 4,4′,4″-Tris(carbazol-9-yl)triphenylamine doped with Tris(2-phenylpyridine) iridium(III) (TCTA:Ir(ppy)_3_ at 8 wt%) and 1,3,5-Tris(1-phenyl-1Hbenzimidazol- 2-yl)benzene doped with Ir(ppy)_3_ (TPBI:Ir(ppy)_3_ at 8 wt%) is used as emitter layer in case of the green OLED. NPB doped with bis(2-methyldibenzo[f,h]quinoxaline)- (acetylacetonate)iridium(III) (NPB: Ir(MDQ)_2_(acac) at 10 wt%) is used as emitter layer in case of a red OLED. The emission layer of each OLED has a thickness of 20 nm. Bis(8-hydroxy-2-methylquinoline)- (4-phenylphenoxy) aluminum (BAlq_2_) is used as a 10 nm thick hole blocking layer. Bathophenanthroline doped with caesium (BPhen:Cs) is used as electron transport layer. The top electrode is made from aluminium forming the cathode.

## Data Availability

The datasets generated during and/or analysed during the current study are available from the corresponding author on reasonable request.
